# US County-Level Variation in Availability and Prevalence of Black Physicians in 1906

**DOI:** 10.1001/jamanetworkopen.2024.10242

**Published:** 2024-05-10

**Authors:** Benjamin W. Chrisinger

**Affiliations:** 1 Department of Social Policy and Intervention, University of Oxford, Oxford, United Kingdom; 2 Now with Department of Community Health, Tufts University, Medford, Massachusetts

## Abstract

**Importance:**

Black physicians are substantially underrepresented in the US health care workforce, with detrimental effects on the health and health care experiences of Black individuals. These contemporary gaps can be traced to the early days of the medical profession using the first edition of the American Medical Directory (AMD).

**Objective:**

To identify state- and county-level patterns related to the training and availability of Black physicians relative to their White counterparts in the 1906 AMD.

**Design, Setting, and Participants:**

For this cross-sectional study, data for 41 828 physician entries in 18 US states in or adjacent to the South as well as the District of Columbia were extracted from the 1906 AMD and aggregated to 1570 counties. Data analysis was performed between September 2023 and January 2024.

**Exposures:**

County-level exposure variables included population density, racial composition, and illiteracy rate among US-born White residents as well as an index of terrain ruggedness and the number of lynchings in the previous decade. Median values of physicians’ distance from place of practice to place of medical training (by race of physician) were also used as an exposure variable.

**Main Outcomes and Measures:**

There were 4 county-level outcomes: (1) presence of any Black physician, (2) proportion of Black physicians per Black population, (3) proportion of White physicians per White population, and (4) community representativeness (reported as the community representativeness ratio). The cross-sectional analysis used generalized additive mixed models with state-level random effects.

**Results:**

Across 1570 counties, Black physicians comprised 746 (1.8%) of the 41 828 physicians in the dataset. Black physicians tended to train further from their place of practice than their White counterparts. The proportion of Black physicians per 1000 Black residents was 0.08 compared with 1.62 for White physicians; these proportions varied substantially by state. At the county level, the presence of any Black physician was associated with percentage Black population (odds ratio [OR], 28.94 [95% CI, 9.77 to 85.76]; *P* ≤ .001), population density (OR, 2.63 [95% CI, 2.03 to 3.40]; *P* ≤ .001), and distance to the nearest Black medical school (OR, 0.62 [95% CI, 0.42 to 0.92]; *P* = .02).

**Conclusions and Relevance:**

A variety of structural disadvantages are illustrated in this cross-sectional study of county-level sociodemographic and geographic characteristics associated with the prevalence of Black physicians in the earliest days of the profession. To demonstrate its broader utility for health disparities research, the dataset has been made publicly available with a visualization platform.

## Key Points

**Question:**

Using physician data from the first American Medical Directory (AMD) published in 1906, which county-level characteristics are associated with the availability and prevalence of Black physicians in the South?

**Findings:**

In this cross-sectional study of 1570 counties, those with lower proportions of Black residents, lower population densities, and greater distances to Black medical schools were more likely to have no Black physicians. Among counties with at least 1 Black physician, higher numbers of Black physicians were associated with lower illiteracy rates among US-born White residents, lower percentage Black population, lower population densities, and shorter median distances to training for Black physicians.

**Meaning:**

These findings suggest that the dataset created in this study may help elucidate key physician- and county-level patterns associated with early racial inequalities in the US medical profession.

## Teaser

This cross-sectional study examines state- and county-level patterns related to the training and availability of Black physicians using data from the first edition of the American Medical Directory.

## Introduction

Today, Black physicians are substantially underrepresented in US medicine, with many researchers pointing to structural barriers that winnow pipelines of would-be Black physicians through economic challenges and experiences of racism.^1,2,3,4^ Researchers have highlighted the critical role of historically Black colleges and universities (HBCUs) in training Black physicians, with some modern HBCUs opening or reopening medical schools to students.^5,6^ The effects of this gap go well beyond professional equality: the undersupply of Black physicians is associated with higher all-cause mortality rates among Black individuals.^7^

These contemporary patterns have deep roots. Scholars have written about the myriad challenges faced by Black physicians in the early 20th century, a period of great change in the medical profession and US society at large. During this time, the American Medical Association (AMA) constructed or was complicit in the use of multiple barriers to keep Black physicians outside mainstream medical institutions, such as refusing to recognize Black delegates at state and national medical society meetings and supporting segregated medical education.^5,8,9,10^ These racist and exclusionary practices added challenges for Black individuals hoping to enter the field of medicine and depressed the numbers of Black physicians.^5,8,10^

One concrete example of the AMA’s anti-Black practices during this period of medical professionalization is in the pages of the American Medical Directory (AMD).^8^ In 1906, the AMA began large-scale efforts to compile and publish a medical directory, listing all credentialed physicians (not just AMA members) by city, county, and state.^11,12^ Many physician records also included information about medical training, graduation year, and specialty. These compendia were intended as a free and authoritative listing of US physicians, “free from the names of quack physicians, fake sanitariums, humbug ‘cures’ and fraudulent medical preparations.”^13^^(p217)^

Black physicians were included in the AMD, with a substantial caveat. Between 1906 and 1940, publishers appended the label “col.” for “colored” to Black physicians’ entries to distinguish them from their White counterparts.^8^ The origins of this practice are unclear, as racial information was not solicited on official biographical information cards distributed by AMA through state and local medical societies.^14^^(p22)^ Available evidence suggests that labels may have been added by county-level officials responsible for compiling and validating local physician lists, and then codified into central AMA recordkeeping (eMethods in Supplement 1). The designation was not without consequence for Black physicians, including further discrimination by insurers and prospective patients.^8,9^ Although researchers eventually used these AMD labels to describe later racial disparities in the medical workforce, this formative period has not been covered, nor have AMD medical school data from this time been used to explore patterns in practice location^15,16^

Although it is understood that underrepresentation has been a problem throughout the history of US medicine, an in-depth perspective on racial disparities in the earliest days of the profession is lacking. Therefore, this study aimed to detail the process of extracting physician-level records from the first AMD and to analyze patterns related to the training and availability of Black physicians. This work also aimed to explore how county-level socioeconomic characteristics and factors related to structural racism were associated with the availability of Black physicians.

## Methods

The University of Oxford Departmental Research Ethics Committee of the Department of Social Policy and Intervention confirmed this cross-sectional study not to be human participant research; thus, informed consent was not required. The study followed the Strengthening the Reporting of Observational Studies in Epidemiology (STROBE) reporting guideline.

### Data Extraction

A specialist document digitization service manually extracted records from a PDF version of the 1906 AMD via double-entry for 18 US states in or adjacent to the South and the District of Columbia (eFigure 1 in Supplement 1). Relevant fields were identified and independently populated by 2 data extractors, including physician name, practice location (city, county, and state), medical school attended and year of graduation, and whether the physician was labeled as Black (no such labels identify other racial or ethnic groups). A small number of institutions were also listed as “colored” in the AMD, although not all physicians who attended these schools were labeled as Black. Thus, a more expansive Black race variable was created to include all graduates of these institutions (n = 113) in addition to those specifically identified in their AMD entry (n = 632) (eTable 1 in Supplement 1). In group-level analyses, physicians who were not classified as Black were assumed to be White. Further details on the data extraction and validation procedure are provided in the eMethods in Supplement 1.

### Practice Location

A geocoding procedure was followed for each physician’s city and county. First, exact matches were identified based on city, county, and state names using National Historical Geographic Information System place shapefiles from 1900 to 1950.^17^ Unmatched cities were then compared with a historical post office database compiled by Blevins and Helbock.^18^ Additionally, geocoding was conducted using OpenStreetMap’s Nominatim API.^19^ For places without an exact match in these databases, a fuzzy matching algorithm was used to identify locations with minor spelling errors or other inconsistencies.^20^ Finally, trained research assistants checked remaining physician records against these databases, using their judgement to correct spelling or formatting errors that prevented exact or fuzzy matching. For physician records that could not be matched to a valid place, county centroids were used, where available.

### Medical Training Information

The 1906 AMD listed medical school locations. For the 37 509 physicians (89.7%) with training information that could be matched to a listed medical school, the straight-line distance between the place of practice and medical school was calculated.^21^ Complete medical training information was missing for 4.7% of Black physicians and 11.1% of White physicians.

### Proportions of Physicians by Population and Race

Population data from the 1910 US Census were used to calculate race-specific values of physicians per person (eg, number of Black physicians divided by Black population), by county (1570 counties total).^17^ Community representativeness ratios (CRRs) were also calculated for each county, providing a straightforward, population-adjusted indicator of racial inequality in representation.^7^ These ratios were calculated by dividing the proportion of Black physicians by the proportion of Black residents in a given county. A value of 1.0 signified that the proportion of Black physicians in a county matched that of Black residents overall. To calculate this measure, nonzero denominators were required (eg, at least 1 Black resident and 1 White resident); 39 counties had no Black residents. Similarly, a CRR was not calculated for the 44 counties with no physicians (n = 44). Ten counties had neither physicians nor any Black residents.

Full-count US Census occupation records from 1910 offered an alternative perspective on AMD-derived prevalence.^22^ To adjust for possible AMD overcounts or undercounts in statistical models, the difference between AMD and US Census counts of physicians (by race) was calculated for each county. Associations between county characteristics associated and possible overcounts or undercounts are described in the eMethods in Supplement 1.

### County Characteristics

Prior literature from this period suggests an association between local area socioeconomic and geographic characteristics and the availability of physicians.^23,24,25^ Data from the 1910 US Census were used to calculate the following relevant variables: percentage Black population, percentage White population, illiteracy rate among US-born White residents (as a measure of socioeconomic disadvantage), and population density.^17,26^ A modern tract-level index of terrain ruggedness was aggregated by 1910 county boundaries, yielding an area-weighted average to represent mountainous or difficult-to-reach places (eFigure 2 in Supplement 1).^27^ To further illuminate structural disadvantages faced by Black physicians, the median distance to medical training for physicians was also calculated for each county, by race; the distance to the nearest Black medical school was also calculated.^24,25^ Population densities and distances to training were log-transformed. Additionally, given the Southern context of anti-Black violence that could have influenced physicians’ practice locations, 2 national lynching databases^28,29,30,31^ were combined to summarize the number of lynchings in each county from 1896 to 1905, the decade immediately before the 1906 AMD.

### Statistical Analysis

Descriptive statistics (proportions and means with SDs) were generated by state of practice across multiple variables, including the median distance to medical training and years of graduation and licensing. The statistical significance of within-state racial differences in the distance between medical training and practice was calculated using *F* tests of equal variances in means.

Physician prevalence outcomes were analyzed using generalized additive mixed models (GAMMs), a type of semiparametric generalized linear model, fit using the *mgcv* package for R.^32^ These models can be fit for both binary and continuous outcomes, specify negative binomial distributions, and accommodate random effects and spatial autocorrelation.^33,34,35,36^ The flexibility of GAMMs and their ability to incorporate geographic information make them a useful tool in these analyses. A series of GAMMs were fit for each outcome with county-level variables, a state-level random effect term, and a smooth term for county centroid coordinates to account for spatial autocorrelation.^37,38^ Smoothing parameters for GAMMs were estimated via restricted maximum likelihood.

Models were fit for 4 distinct outcomes. Variable coefficients and magnitudes were not intended to be directly comparable between these outcomes, although differences in significance and direction of association were of interest. Two outcomes examined different patterns in the locations of Black physicians: (1) the complete absence of Black physicians and (2) the undersupply of Black physicians. A binary outcome (0 vs any Black physicians) was examined with a binary logistic regression using the full dataset. Then, a negative binomial model was fit for the number Black physicians, adjusted for the number of Black residents with an offset term (log[No. Black residents + 1]), using only counties with at least 1 Black physician (320 counties). For indirect comparison, negative binomial models were also fit for the count of White physicians with a White population offset term, using only counties with at least 1 White physician (1526 counties). Counties with extreme prevalence values (ie, >10 physicians/1000 residents) were excluded (7 counties, extreme prevalence of Black physicians; 3 counties, extreme prevalence of White physicians). Finally, GAMMs were fit with CRRs as a continuous outcome using a Gaussian distribution. Here, only counties with a CRR greater than 0 were initially included (320 counties), as the dimension of no representation was already explored in the binary logistic regression; of these, 8 counties were also excluded where Black residents represented less than 2.0% of the population, which yielded extreme CRR values (ie, CRR >10.0). Washington, DC, and 9 counties with missing variables were excluded from statistical analyses.

All variables were assessed for multicollinearity using the variance inflation factor, and the overall fit of models to the dataset was assessed using the Akaike information criterion. Coefficient estimates and 95% CIs were calculated to identify associations detectable at the α = .05 level. Analyses were performed using R, version 4.0.5 (R Project for Statistical Computing).^37^ Data analysis was performed between September 2023 and January 2024.

## Results

### Physician Prevalence and Characteristics by State

Of the 41 828 physician records across 18 states and the District of Columbia extracted from the 1906 AMD, 746 physicians (1.8%) were classified as Black (Table 1). Across the dataset, the proportion of physicians per 1000 residents was 1.22. This value did not hold when adjusted for population and race; for instance, Mississippi had more than 2.20 White physicians per 1000 White residents, yet only 0.02 Black physicians per 1000 Black residents. Overall, the proportion of Black physicians per 1000 Black residents was 0.08 compared with 1.62 for White physicians. These state-level disparities were also reflected in the CRR, with Mississippi being one of the least racially representative states in the South (CRR = 0.02).

**Table 1.** Southern Physicians in the 1906 American Medical Directory by Race and State

State	Physicians		Residents^a^		Physician availability^b^		CRR
	All, No.	Black, No. (%)	All, No.	Black, No. (%)	Black proportion	White proportion	
Alabama	2115	40 (1.9)	2 138 093	908 282 (42.5)	0.04	1.69	0.04
Arkansas	2324	57 (2.5)	1 574 449	442 891 (28.1)	0.13	2.00	0.09
Delaware	222	1 (0.5)	202 322	31 181 (15.4)	0.03	1.29	0.03
DC	1019	3 (0.3)	331 069	94 446 (28.5)	0.03	4.30	0.01
Florida	617	25 (4.1)	752 619	308 669 (41)	0.08	1.33	0.10
Georgia	2768	55 (2)	2 609 121	1 176 987 (45.1)	0.05	1.89	0.04
Kansas	2401	16 (0.7)	1 690 949	54 030 (3.2)	0.30	1.46	0.21
Kentucky	3774	108 (2.9)	2 289 905	261 656 (11.4)	0.41	1.81	0.25
Louisiana	1548	42 (2.7)	1 656 388	713 874 (43.1)	0.06	1.60	0.06
Maryland	1780	6 (0.3)	1 295 346	232 250 (17.9)	0.03	1.67	0.02
Mississippi	1757	24 (1.4)	1 797 114	1 009 487 (56.2)	0.02	2.20	0.02
Missouri	5922	28 (0.5)	3 293 335	157 452 (4.8)	0.18	1.88	0.10
North Carolina	1529	48 (3.1)	2 206 287	697 843 (31.6)	0.07	0.99	0.10
Oklahoma	1884	22 (1.2)	1 657 155	137 612 (8.3)	0.16	1.29	0.14
South Carolina	1019	22 (2.2)	1 515 400	835 843 (55.2)	0.03	1.47	0.04
Tennessee	2962	89 (3.0)	2 184 789	473 088 (21.7)	0.19	1.68	0.14
Texas	4821	84 (1.7)	3 896 542	690 049 (17.7)	0.12	1.48	0.10
Virginia	1961	55 (2.8)	2 061 612	671 096 (32.6)	0.08	1.37	0.09
West Virginia	1405	21 (1.5)	1 221 119	64 173 (5.3)	0.33	1.20	0.28
Overall	41 828	746 (1.8)	34 373 614	8 960 909 (26.1)	0.08	1.62	0.07

Abbreviation: CRR, community representativeness ratio.

^a^ Total population of White and Black residents according to the 1910 US Census.

^b^ Defined as number of physicians divided by 1000 residents, by race. White physicians were defined as those not identified as Black.

Physicians traveled a mean (SD) of 462.5 (481.2) km for medical training, with differences by state of practice (Table 2). In most states, Black physicians tended to train further away than their White counterparts. However, 2 states had the opposite pattern: Black physicians in Tennessee and North Carolina were substantially closer to their medical schools compared with White physicians. Medical schools in Tennessee trained 52.3% of the Black physicians in the dataset, and those in North Carolina trained 16.0%. Several Southern states trained no Black physicians; for instance, Alabama, Arkansas, and South Carolina had no Black trainees, although they trained sizeable numbers of White physicians (785, 389, and 530 graduates, respectively). Smaller numbers of Black physicians in this dataset trained outside the South (53 in Washington, DC, and 52 in other states). These patterns are consistent with descriptive differences between counties according to levels of racial representativeness (Table 3).

**Table 2.** Racial Differences in Distance to Medical Training for Southern Physicians in the 1906 American Medical Directory Subset

State	All physicians, No.^a^	Black physicians, No. (%)	Distance, km		*P* value^b^
			Mean (SD)	Black-White difference	
Alabama	1920	39 (2.0)	589.7 (425.6)	161.1	.006
Arkansas	1701	54 (3.2)	640.2 (335.4)	131.9	.02
Delaware	216	1 (0.5)	159.3 (NA)	25.8	NA
DC	995	3 (0.3)	731.6 (309.5)	637.8	<.001
Florida	513	24 (4.7)	910.6 (232.4)	−77.1	.39
Georgia	2559	53 (2.1)	572.0 (232.3)	251.6	<.001
Kansas	2208	16 (0.7)	872.7 (146.8)	129.0	.36
Kentucky	3619	105 (2.9)	196.8 (201.2)	−5.3	.83
Louisiana	1357	38 (2.8)	454.1 (552.3)	−10.9	.91
Maryland	1608	3 (0.2)	680.4 (283.6)	598.8	<.001
Mississippi	1393	23 (1.7)	576.6 (325.6)	96.1	.19
Missouri	5253	25 (0.5)	712.5 (445.6)	393.2	<.001
North Carolina	1459	46 (3.2)	211.0 (208.9)	−276.9	<.001
Oklahoma	1600	18 (1.1)	970.1 (241.2)	88.7	.37
South Carolina	1000	22 (2.2)	528.1 (295.5)	146.0	.03
Tennessee	2533	86 (3.4)	162.9 (194.6)	−103.5	.003
Texas	4268	79 (1.9)	1091.2 (398.0)	39.2	.52
Virginia	1924	54 (2.8)	247.0 (144.7)	−7.1	.81
West Virginia	1249	18 (1.4)	424.4 (186.9)	38.8	.39
Overall	37375	707 (1.9)	505.1 (429.6)	43.4	.02

Abbreviation: NA, not applicable.

^a^ Only includes physicians with complete medical training information.

^b^ *F* tests of equal variances in means by race and state.

**Table 3.** County Characteristics by CRR Tertile^a^

Characteristic	Counties with no Black physicians (n = 853)	Counties by CRR tertile		
		Lowest, least representative (n = 107)	Middle (n = 106)	Highest, most representative (n = 98)
CRR	0	0.08 (0.03)	0.18 (0.03)	0.41 (0.21)
Distance to medical school, km, median (IQR)				
For Black physicians	NA^b^	498 (263.9-755.4)	385 (193.6-752.0)	372 (192.2-757.0)
Closest Black medical school	299 (174.7-488.2)	284 (187.0-432.0)	235 (118.2-493.3)	246 (129.0-583.0)
Proportion of Black physicians per 1000 Black residents	0	0.1 (0.06)	0.25 (0.18)	0.57 (0.36)
Percentage Black population	0.29 (0.23)	0.48 (0.21)	0.37 (0.20)	0.20 (0.16)
Percentage illiterate population	0.08 (0.06)	0.07 (0.06)	0.06 (0.05)	0.07 (0.06)
Population density, 1000 residents/km^2^	19.8 (159.6)	140 (802.1)	88.9 (471.2)	70.4 (307.3)
Terrain ruggedness index	24.1 (23.6)	16.2 (14.6)	15.2 (13.0)	25.7 (24.8)
No. of lynchings (1886-1905)	0.27 (0.45)	0.5 (0.50)	0.42 (0.50)	0.18 (0.39)

Abbreviations: CRR, community representativeness ratio; NA, not applicable.

^a^ Unless specified otherwise, values are presented as means (SDs). Counties where the CRR equals 0 (no Black physicians) are presented separately to illustrate intratertile variability. Data exclude counties where the percentage Black population was less than 2.0% (386 counties).

^b^ Information could not be generated for counties without Black physicians.

### County Characteristics and Physician Prevalence and Representativeness Outcomes

Four outcomes are reported here (Table 4). The presence of any Black physician was positively associated with percentage Black population (odds ratio [OR], 28.94 [95% CI, 9.77-85.76]) and population density (OR, 2.63 [95% CI, 2.03 to 3.40]) and was negatively associated with distance to the nearest Black medical school (OR, 0.62 [95% CI, 0.42 to 0.92]). Among counties with at least 1 Black physician, higher numbers of Black physicians were associated with lower illiteracy rates among US-born White residents (incidence rate ratio [IRR], 0.01 [95% CI, 0-0.05]), lower population densities (IRR, 0.83 [95% CI, 0.76-0.91]), lower percentage Black populations (IRR, 0.10 [95% CI, 0.06-0.17]), and shorter median distances to training for Black physicians (IRR, 0.91 [95% CI, 0.86-0.97]). In counties with at least 1 White physician, the number of White physicians was associated with higher population densities (IRR, 1.12 [95% CI, 1.09-1.15]) and with lower illiteracy rates (IRR, 0.07 [95% CI, 0.05-0.12]) and lower percentage White populations (IRR, 0.32 [95% CI, 0.28-0.36]). Community representativeness was positively associated with the median distance to training for White physicians (β coefficient, 0.02 [95% CI, 0-0.04]) and negatively associated with instances of lynching between 1886 and 1905 (β coefficient, −0.01 [95% CI, −0.02 to −0]). Additional model fits are provided in eTables 2 to 5 in Supplement 1. Sensitivity analyses are reported in the eMethods, eResults, and eTable 6 in Supplement 1.

**Table 4.** Results of Generalized Additive Mixed Models Associating County Characteristics With Physician Prevalence by Race and Racial Representativeness Outcomes^a^

Variable	Physician prevalence outcome			
	Presence of any Black physician, OR (95% CI)^b^	Prevalence, IRR (95% CI)		CRR, β coefficient (95% CI)
		Black physicians^b^	White physicians^c^	
Percentage Black population	28.94 (9.77 to 85.76)	0.10 (0.06 to 0.17)	NA^d^	NA
Percentage White population	NA	NA	0.32 (0.28 to 0.36)	NA
Percentage illiterate population^e^	0.11 (0 to 4.69)	0.01 (0 to 0.05)	0.07 (0.05 to 0.12)	−0.07 (−0.47 to 0.33)
Terrain ruggedness index^f^	0.80 (0.57 to 1.13)	1.01 (0.87 to 1.17)	0.98 (0.94 to 1.02)	0.04 (−0 to 0.08)
Population density^f^	2.63 (2.03 to 3.40)	0.83 (0.76 to 0.91)	1.12 (1.09 to 1.15)	−0.01 (−0.03 to 0.02)
No. of lynchings (1886-1905)	1.06 (0.96 to 1.18)	0.95 (0.90 to 1.01)	1.00 (0.99 to 1.01)	−0.01 (−0.02 to −0)
Distance to nearest Black medical school^f^	0.62 (0.42 to 0.92)	NA	NA	NA
Distance to training, median^f^^,^^g^				
Black physicians	NA	0.91 (0.86 to 0.97)	NA	−0.02 (−0.04 to 0)
White physicians	NA	NA	1.01 (0.99 to 1.03)	0.02 (0 to 0.04)
No. of observations	1569	310	1518	307

Abbreviations: CRR, community representativeness ratio; IRR, incidence rate ratio; NA, not applicable; OR, odds ratio.

^a^ All models include state-level random effect and adjustment for spatial position.

^b^ Model included offset term: log(total Black population + 1).

^c^ Model included offset term: log(total White population + 1).

^d^ Cells with NA denote cases where variables were not included in a particular model.

^e^ Illiteracy among US-born White population.

^f^ Log-transformed.

^g^ Median value.

### Data Visualization

A visualization tool, the Deep Roots of Racial Inequalities in US Healthcare,^39^ was developed to allow public engagement with the dataset used in this study (Figure). Users can (1) filter the data based on race, practice, and medical school location, (2) explore how physician characteristics differed across the South, and (3) download the dataset.

**Figure.**
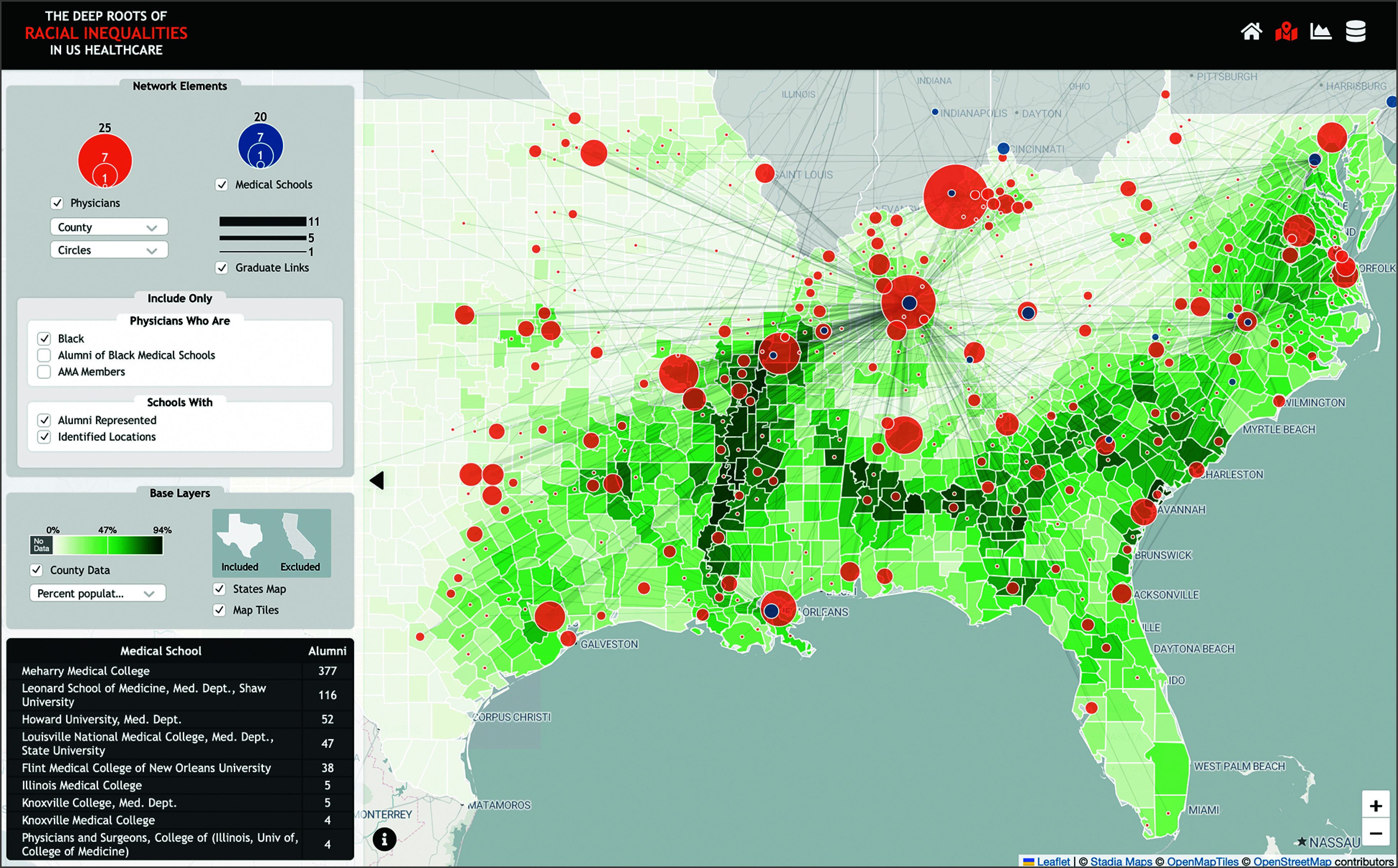
Interactive Data Visualization Website The Deep Roots of Racial Inequalities in US Healthcare tool is available online.^39^ Visitors can filter physician-level data by race and place of practice, visualize linkages to places of training with county demographic information, and download the dataset.

## Discussion

Physician-level data were extracted from the first AMD and analyzed to identify physician and county characteristics associated with the prevalence of Black physicians in 1906. The findings of this study point to several patterns. In terms of training, Black physicians tended to travel further from their place of practice than their White counterparts (although this varied substantially by state) and were predominantly trained at Southern institutions. Proportions of Black physicians to Black populations also were substantially below those for White populations, with notable geographic variation.

In this study, counties with lower percentage Black population, lower population densities, and greater distances to Black medical schools were more likely to have no Black physicians. Greater numbers of Black physicians were associated with lower illiteracy rates among US-born White residents, lower percentage Black population, lower population densities, and shorter median distances to training for those Black physicians. More White physicians could be found in counties where population density and diversity were higher and illiteracy rates were lower. The negative association of lynching with racial representativeness was also detectable, with lower representativeness in counties with more of these violent attacks. These patterns, although not causally investigated in this study, suggest how social structures, including violence, may help explain the patterning of inequality beyond sociodemographics and geography alone.

Previous large-scale analyses of historical patterns in Black medical training and practice have been limited, in part, by the lack of accessible AMD data. Beginning in the 1930s, *JAMA* published periodic snapshots of the Black medical workforce; although some have offered similar assessments of training and practice disparities for later time periods, none have replicable or searchable datasets for contemporary researchers. In 1932, Lewis^15^ used the AMD to estimate the proportion of the Black population per Black physician to be 2988 nationally and 3056 across Southern states, far worse than the national figure of 724 for White populations. In 1942, Cornely^16^ called attention to decreasing numbers of Black physicians (compared with 1932), especially across the Southern states, which then had 4489 Black residents per Black physician. In 1969, Haynes,^40^ using 1967 data from the National Medical Association (NMA) and the AMA, found that only 2% of American physicians were Black; this figure is remarkably close to our estimate of 1.8%, although notably focusing on a national rather than Southern population. Our 1906 data make clear how poorly Black individuals were reflected in medicine during earlier periods: across the same Southern states, there were 12 175 Black residents per Black physician compared with 822 for White populations. Notably, this helps illustrate both the progress made in racial representation (a 4-fold improvement between 1906 and 1932), but also the depth and scale of the initial racial disparity.

Lewis^15^ similarly noted the importance of Southern medical schools for addressing the need for Black physicians and the wide variability in population-adjusted rates of physicians by race. Haynes^40^ also highlighted the primacy of Meharry Medical College and Howard University in training Black physicians (83%), a pattern observed in our 1906 dataset to a lesser degree (57%). Critically, 29% of Black physicians in our dataset attended schools that would be closed following the 1910 Flexner report; as others have noted, the effects of these closures continue to be felt today.^5^

### Limitations

This study has several limitations. First, although the AMD labeled Black physicians, there was no label for other racial or ethnic groups. Thus, the number of physicians labeled as White is likely to be an overcount. Relatedly, the AMD is not a perfect record of all Black physicians. The reporting of race to directory publishers is likely to be an imperfect proxy. Indeed, mismatches with US Census occupation records (noted in eTable 6 in Supplement 1), as well as the risks to physicians publicly labeled as Black, provide some basis for assuming that an undercount is likely.^8,9^ Still, the NMA reported fewer than 50 members in 1904, and 264 members in 1907 (although a “mailing list” of 1227 names is noted).^41^ Although definitive answers to questions of racial labeling and “passing” are beyond the scope of this work,^42,43^ US Census occupation data help to adjust for the possible influence of overcounting and undercounting.

This analysis focused on the South and adjacent states. This pragmatic decision has grounding in the social and demographic context of 1906, when the majority of the nation’s Black population lived in Southern states.^25^ Subsequent analyses noted Black physicians moving to Northern and Western states, part of the larger Great Migration to escape the Jim Crow South.^15,16^ Still, this subnational focus limits a more nuanced examination of these disparities. Relatedly, the cross-sectional nature of the dataset precludes analyses of how disparities changed over time.

## Conclusions

Black physicians in the early 20th century South faced a litany of challenges to obtain a medical education and establish a medical practice.^8,9,10,25,44^ Yet, many persisted. The dataset created in this cross-sectional study and the analyses presented herein are a testament to the structural racism experienced by early Black physicians, and these findings give greater detail to the conditions and contexts in which they studied and practiced.

## Notes

Supplement 1.
eMethods.
**eFigure 1.** Extract From a Page of the 1906 American Medical Directory **eFigure 2.** Terrain Ruggedness Index
eResults.

eReferences
**eTable 1.** Summary of Black Physician Counts Using Different Racial Categorization Systems **eTable 2.** Comparisons of Different Generalized Additive Mixed Model Specifications: Presence of Black Physicians (Binary Logistic) **eTable 3.** Comparisons of Different Generalized Additive Mixed Model Specifications: Black Physicians per Black Population (Negative Binomial) **eTable 4.** Comparisons of Different Generalized Additive Mixed Model Specifications: White Physicians per White Population (Negative Binomial) **eTable 5.** Comparisons of Different Generalized Additive Mixed Model Specifications: Community Representativeness Ratio (CRR; Continuous) **eTable 6.** Associations Between County Characteristics and Differences Between 1906 American Medical Directory and 1910 US Census Occupational Records

Supplement 2.
Data Sharing Statement
