# Supplementary material for: US County-Level Variation in Availability and Prevalence of Black Physicians in 1906
Source: JAMA Netw Open. 2024 May 10;7(5):e2410242. doi: 10.1001/jamanetworkopen.2024.10242 (PMC11087833; doi:10.1001/jamanetworkopen.2024.10242)
Supplement: Supplement 1. — eMethods. eFigure 1. Extract From a Page of the 1906 American Medical Directory eFigure 2. Terrain Ruggedness Index eResults. eReferences eTable 1. Summary of Black Physician Counts Using Different Racial Categorization Systems eTable 2. Comparisons of Different Generalized Additive Mixed Model Specifications: Presence of Black Physicians (Binary Logistic) eTable 3. Comparisons of Different Generalized Additive Mixed Model Specifications: Black Physicians per Black Population (Negative Binomial) eTable 4. Comparisons of Different Generalized Additive Mixed Model Specifications: White Physicians per White Population (Negative Binomial) eTable 5. Comparisons of Different Generalized Additive Mixed Model Specifications: Community Representativeness Ratio (CRR; Continuous) eTable 6. Associations Between County Characteristics and Differences Between 1906 American Medical Directory and 1910 US Census Occupational Records [file jamanetwopen-e2410242-s001.pdf]

Supplementary Online Content

Chrisinger BW. US county-level variation in availability and prevalence of Black physicians in 1906. *JAMA Netw Open*. 2024;7(5):e2410242. doi:10.1001/jamanetworkopen.2024.10242

eMethods.

eFigure 1. Extract From a Page of the 1906 American Medical Directory

eFigure 2. Terrain Ruggedness Index

eResults.

eReferences

eTable 1. Summary of Black Physician Counts Using Different Racial Categorization Systems

eTable 2. Comparisons of Different Generalized Additive Mixed Model Specifications: Presence of Black Physicians (Binary Logistic)

eTable 3. Comparisons of Different Generalized Additive Mixed Model Specifications: Black Physicians per Black Population (Negative Binomial)

eTable 4. Comparisons of Different Generalized Additive Mixed Model Specifications: White Physicians per White Population (Negative Binomial)

eTable 5. Comparisons of Different Generalized Additive Mixed Model Specifications: Community Representativeness Ratio (CRR; Continuous)

eTable 6. Associations Between County Characteristics and Differences Between 1906 American Medical Directory and 1910 US Census Occupational Records

This supplementary material has been provided by the authors to give readers additional information about their work.

© 2024 Chrisinger BW. *JAMA Network Open.*

eMethods.

S1.1. Data extraction

Extracting physician records from the historical AMDs to conduct modern analyses is difficult for several reasons. Multiple public archives have published versions of the AMD as digitized PDFs.[1,2] These sources varied widely in terms of optical quality, though in some instances, information could be extracted using digital text scraping tools.[3] A separate challenge is the Directory's formatting; each page is arranged as a series of three columns, which effectively block many of the known PDF text extraction tools. Additionally, though entries are generally consistent in terms of the information presented, some deviations do occur, which challenges a fully automated process of extracting individual records from scraped text.

Given these challenges in automating data extraction, a specialist document digitization service was employed to manually extract records via double-entry for a subset of 18 Southern and adjacent states. Relevant fields were identified and independently populated by two individual data extractors, including physician name, practice location (city, county, and state), medical school attended and year of graduation, and whether the physician was labelled as Black. While the AMD identified Black physicians, there was no such labelling system for other racial or ethnic groups. In group-level analyses (subsequently described), doctors who were not classified as Black were assumed to be White, though a label of "White" was not appended to physician records in the dataset.

From the records of medical training, we know that small numbers of physicians were trained outside the US; of these, most trained in Europe or Canada, though small numbers also reported training in Mexico and Cuba. While this provides a limited understanding of other racial or ethnic groups in the AMD, it suggests that the number of "White" doctors in the dataset is likely to be a slight overcount.

eFigure 1 shows an example image from the Directory of two physician records. Information given about the first physician (Hitchcock) includes year of birth (1866), school (University of Louisville), graduation and license year (both 1893), and AMA membership. The second physician (Maclin) is identified as Black ("col."), attended Meharry Medical College, graduated, and was licensed in 1905.

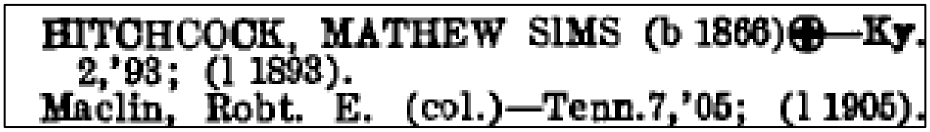
HITCHCOCK, MATHEW SIMS (b 1866)⊕—Ky. 2,'93; (l 1893).
Maclin, Robt. E. (col.)—Tenn.7,'05; (l 1905).

eFigure 1. Extract from a page of the 1906 American Medical Directory.[2]

A small number of institutions were also described as "colored" in the AMD: Louisville National Medical College, Flint Medical College of New Orleans University, Leonard School of Medicine of Shaw University, Knoxville Medical College, University of West Tennessee, and Meharry Medical College. Not all physicians who attended these schools were identified as Black in the AMD; thus, a more expansive Black race variable was created to include all graduates of these institutions (n=113) in addition to those specifically identified in their AMD entry (n=632). Howard University, known in modern times as a prominent HBCU, admitted students of all races, and therefore was not used in this reclassification.[4] **eTable 1** describes state-level differences in Black physician counts according to these classification approaches.

The questions of where racial labelling originated and how it was implemented are more challenging. The Standard Directory, purchased by the AMA as the foundational Directory dataset, did not include racial designations.[5] "Biographical cards" requested by AMA and state medical societies (in the South and beyond), and published in the pages of *JAMA* and state medical journals, did not request racial details.[6] Some county-level societies similarly published blank card forms, hoping that physicians would help update their records; an example of this from Arkansas includes nothing about race.[7(p22)] Digitized biographical cards – now available as the "Deceased Physicians File" – provides further clues but no answers.[8] The "(col.)" label appears on many Black physicians' cards, though at least example suggests these labels were added after 1906 and based on information gleaned from state society publications.[9,10]

Taken together, this seems to suggest a local origin for the labels, which were centrally codified by AMA. Local physicians were likely to be to known to county medical society secretaries, who were charged with collecting and

© 2024 Chrisinger BW. *JAMA Network Open.*

verifying Directory records.[11] It is possible that these officials – in the South and beyond – added labels based on their perception of physicians' race. While the question of racial perceptions is one that others have explored in the context of other historical datasets (e.g., Census enumerators), a conclusive answer is beyond the scope of this paper. Still, the expanded racial classification used in this paper (physicians with the label or those who attended Black medical schools) addresses some of the uncertainty surrounding the label.

S1.2. Database validation
A 10% random sample of directory pages (n=25) were selected for auditing by trained research assistants to verify physician records (n=3528) including their location, medical school information, and racial classification status. No errors were found in location or medical school information, and only minor differences were identified in the racial classification (1 "(col.)" identifier occurred in a non-standard place and was not originally identified as Black; another was misclassified as Black due to the string "col." being used as an abbreviation of the word "college"). Additionally, all physicians classified as Black in the database were cross-referenced in the original directory document. A further 322 physician records were hand-checked if they reported attending a predominantly Black-serving medical institution, but were not otherwise classified as Black.

S1.3. Exclusions
A small number of physician entries were excluded from analyses: physicians listed as "not in practice" (n=116), and those who attended "fraudulent" medical institutions (n=27). Of these exclusions, Black doctors only comprised 1.5% of those listed as not in practice (n=7).

S1.4. Terrain Ruggedness Index
To represent how some counties may have been physically difficult to access, a modern measure of terrain ruggedness is used.[12] While this modern measure could differ from the terrain of the study period, the index primarily captures the mountainous regions of the South (see **eFigure 2**), rather than small-area differences (e.g., levelling of surfaces for urban development).

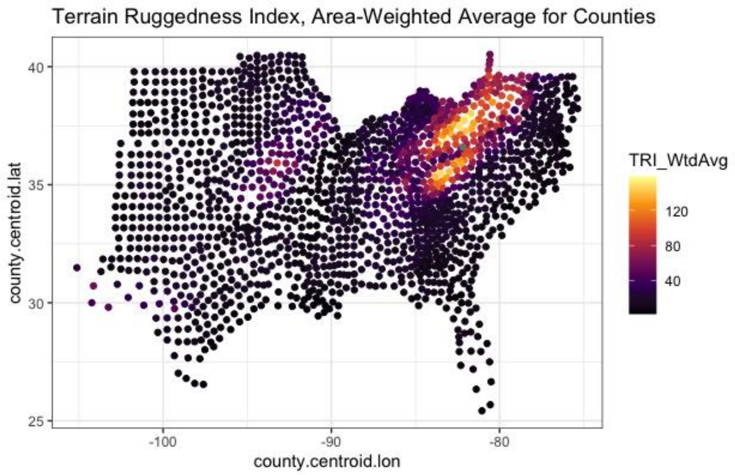

eFigure 2. Terrain Ruggedness Index

S1.5. Sensitivity analyses
Census records of employment provide another alternative data source for exploring the distribution and characteristics of physicians by race. Full-count Census records from 1900 and 1910, for instance, can reveal where Black and White physicians resided (aggregated by county).[13] Notably, these residence-based records could differ from places of practice. Additionally, these self-reported occupation records could be subject to the same kinds of quality biases for which Polk's Register was criticized. Most critically, these records provide no information on the places or years of medical training, features that enable the AMA Directory dataset to help illustrate issues in the pipeline of medical education.

Despite the shortcomings of the Census occupational records, they offer a useful means of triangulating the patterns observed in the AMA Directory dataset. An analogous inequality measure was constructed using 1910 Census counts of physicians by race. Thus, an indicator of potential under/overcounts of White or Black physicians in the AMA dataset was used to filter data in descriptive statistics and included as a covariate in statistical models to help mitigate the effects of potential discrepancies. To further consider the kinds of places that may be especially subject to discrepancies between the AMA Directory and Census records, multivariate linear regressions were performed to examine how differences in the count of White and Black doctors varied according to county characteristics.

eResults.

S2.1 Sensitivity analyses

Counties' proportions of Black residents (ß Estimate=1.09, SE=0.51) and population densities (ß Estimate =1.41, SE=0.10) were positively associated with AMD Black doctor counts that were lower than those from the Census. Negative associations were observed between illiteracy rate (ß Estimate =-5.72, SE=1.67) and distance to training (ß Estimate =-0.55, SE=0.11). For White doctor counts, AMD undercounts (relative to Census figures) were more likely in counties with larger population densities (ß Estimate =6.41, SE=0.48) and proportions of White residents (ß Estimate =10.10, SE=2.47), and lower illiteracy rates (ß Estimate =-19.43, SE=8.14). **eTable 6** provides further details of these models.

While it is beyond the scope of this paper to discern whether these discrepancies are an artefact of differences in construct (i.e., place of practice versus place of residence) or timing (i.e., 1906 versus 1910), or are true under/over-counts in the AMA Directory, these detectable associations are worth noting for researchers who may use the dataset in their own analyses. Further research, especially archival and historical analyses, may help illuminate more about the racial labelling practices in the AMD, and how these interface with individuals who could "pass" as White.[14–18]

© 2024 Chrisinger BW. *JAMA Network Open.*

eReferences

1. Robinson WJ. The American Medical Directory. *Journal of the American Medical Association*. 1906;XLVII(3):217. doi:10.1001/jama.1906.02520030059013

2. American Medical Association. *American Medical Directory.* American Medical Association etc.; 1906. Accessed April 28, 2023. https://catalog.hathitrust.org/Record/000543547

3. Smith SM. Digitizing Doctors: Methodologies for Creating a Database from Historical Directories of Physicians. *Med Hist*. 2017;61(4):611-614. doi:10.1017/mdh.2017.71

4. Lloyd SM. History | Howard University College of Medicine. Howard University College of Medicine History. Published May 2006. Accessed September 25, 2023. https://medicine.howard.edu/about/history

5. G.P. Englehard & Company. *The Standard Medical Directory of North America, 1903-4*. G.P. Englehard & Company; 1904. Accessed January 24, 2024. https://hdl.handle.net/2027/uc1.31378008164686?urlappend=%3Bseq=5

6. BIOGRAPHICAL CARD INDEX AND DIRECTORY. *Journal of the American Medical Association*. 1905;XLV(21):1574-1575. doi:10.1001/jama.1905.02510210044006

7. Arkansas Medical Society. *Monthly Bulletin of the Arkansas Medical Society*. Arkansas Medical Society; 1906. Accessed January 24, 2024. http://archive.org/details/monthlybulletino2190unse

8. National Library of Medicine. Genealogical Research in the History of Medicine- American Medical Association (AMA) Deceased Physicians Card File. Accessed April 17, 2024. https://www.nlm.nih.gov/hmd/genealogy/ama-deceased-physicians.html

9. Medical Association of the State of Alabama. *Transactions of the Medical Association of the State of Alabama*. Montgomery, Ala. [etc.]; 1930. Accessed February 12, 2024. http://archive.org/details/transactionsmed17alabgoog

10. American Medical Association. United States Deceased Physician File, 1864-1968. https://familysearch.org/ark:/61903/3:1:3QS7-99QP-VQNN?cc=2061540&wc=M6YD-WW5%3A353124801

11. Texas Medical Association. *Texas State Journal of Medicine*. Austin : Texas Medical Association; 1906. Accessed April 17, 2024. http://archive.org/details/texasstatejourna1110texa

12. Dobis EA, Cromartie J, Williams R, Reed K, United States. Department of Agriculture. Economic Research Service,. *Characterizing Rugged Terrain in the United States*. Economic Research Service, U.S. Department of Agriculture; 2023. doi:10.32747/2023.8134137.ers

13. Ruggles S, Fitch CA, Goeken R, et al. IPUMS Ancestry Full Count Data. Published online 2021. doi:https://doi.org/10.18128/D014.V3.0

14. Chachere KA. *Visually White, Legally Black: Miscegenation, the Mulatto, and Passing in American Literature and Culture, 1865–1933*. Illinois State University; 2004. Accessed December 9, 2023. https://search.proquest.com/openview/14fae6400d2a1d306552bd5498243d48/1?pq-origsite=gscholar&cbl=18750&diss=y&casa_token=iHp06Z5QQCkAAAAA:s7AnqZoDyP5_ttKppfLJAmr6dB36sSorOys2wPme--HWXBg3fLmiz1BRTn7-Xv8psgsFOvPratE

15. Womack GC. *From" Mulatto" To" Negro": How Fears of'Passing'Changed the 1930 United States Census*. Simmons College; 2017. Accessed December 9, 2023. https://search.proquest.com/openview/eba2b3153629faed1c7c42ab3cc49326/1?pq-origsite=gscholar&cbl=18750&casa_token=VkhsRA5sspAAAAAA:NVw5ccZl-nmR_hyoTSIgCn1gJLoZyYbDKxeb6sos55HPTyghJw_1zQuWYI3bcufkki5sDufgCVc

16. Toplin RB. Between Black and White: Attitudes Toward Southern Mulattoes, 1830-1861. *The Journal of Southern History*. 1979;45(2):185-200. doi:10.2307/2208151

17. Bodenhorn H. The Mulatto Advantage: The Biological Consequences of Complexion in Rural Antebellum Virginia. *The Journal of Interdisciplinary History*. 2002;33(1):21-46. Accessed February 5, 2024. https://www.jstor.org/stable/3656920

18. Zackodnik T. Fixing the Color Line: The Mulatto, Southern Courts, and Racial Identity. *American Quarterly*. 2001;53(3):420-451. Accessed February 5, 2024. https://www.jstor.org/stable/30041900

© 2024 Chrisinger BW. *JAMA Network Open.*

eTable 1. Summary of Black Physician Counts Using Different Racial Categorization Systems

	All doctors	AMD-identified Black doctors (%)	AMD-identified Black doctors or Black medical school graduate (%)
Alabama	2115	36 (5.7)	40 (5.36)
Arkansas	2324	49 (7.75)	57 (7.64)
Delaware	222	1 (0.16)	1 (0.13)
District of Columbia	1019	2 (0.32)	3 (0.4)
Florida	617	25 (3.96)	25 (3.35)
Georgia	2768	51 (8.07)	55 (7.37)
Kansas	2401	2 (0.32)	16 (2.14)
Kentucky	3774	93 (14.72)	108 (14.48)
Louisiana	1548	36 (5.7)	42 (5.63)
Maryland	1780	4 (0.63)	6 (0.8)
Mississippi	1757	19 (3.01)	24 (3.22)
Missouri	5922	25 (3.96)	28 (3.75)
North Carolina	1529	46 (7.28)	48 (6.43)
Oklahoma	1884	18 (2.85)	22 (2.95)
South Carolina	1019	21 (3.32)	22 (2.95)
Tennessee	2962	68 (10.76)	89 (11.93)
Texas	4821	69 (10.92)	84 (11.26)
Virginia	1961	49 (7.75)	55 (7.37)
West Virginia	1405	18 (2.85)	21 (2.82)

Abbreviations: AMD, American Medical Directory

© 2024 Chrisinger BW. *JAMA Network Open.*

eTable 2. Comparisons of Different Generalized Additive Mixed Model Specifications: Presence of Black Physicians (Binary Logistic)

	Dependent variable:		
	bin.black.doc		
	Model 1 (Variables Only)	Model 2 (+ State-Level Random Effect)	Model 3 (+ Spatial Coordinates)
	(1)	(2)	(3)
% Black population	0.949	2.581	3.365
	(0.312, 1.585)	(1.679, 3.483)	(2.280, 4.451)
	p = 0.004***	p = 0.00000***	p = 0.000***
% Illiterate population	-4.730	-3.315	-2.248
	(-7.832, -1.628)	(-6.798, 0.167)	(-6.037, 1.542)
	p = 0.003***	p = 0.063*	p = 0.246
Log(Terrain ruggedness index)	-0.426	-0.378	-0.224
	(-0.646, -0.206)	(-0.645, -0.112)	(-0.569, 0.121)
	p = 0.0002***	p = 0.006***	p = 0.204
Log(Population density)	0.708	0.918	0.965
	(0.495, 0.921)	(0.684, 1.151)	(0.708, 1.222)
	p = 0.000***	p = 0.000***	p = 0.000***
Number of lynchings, 1896-1905	0.078	0.061	0.062
	(-0.017, 0.174)	(-0.039, 0.161)	(-0.041, 0.164)
	p = 0.109	p = 0.233	p = 0.238
Log(Distance to nearest Black medical school)	-0.470	-0.461	-0.481
	(-0.679, -0.262)	(-0.771, -0.151)	(-0.876, -0.086)
	p = 0.00002***	p = 0.004***	p = 0.018**
Difference AMD – Census Black doctor count	0.105	0.069	0.059
	(0.036, 0.174)	(-0.001, 0.138)	(-0.011, 0.129)
	p = 0.003***	p = 0.053*	p = 0.098*
Constant	0.546	-0.796	-1.475
	(-1.184, 2.277)	(-3.049, 1.456)	(-4.106, 1.155)
	p = 0.537	p = 0.489	p = 0.272
Observations	1,569	1,569	1,569
Adjusted R^2	0.184	0.246	0.264
Log Likelihood	-664.540	-632.662	-624.154
UBRE	665.997	641.810	637.888
Note:	*p<0.1; **p<0.05; ***p<0.01		

© 2024 Chrisinger BW. *JAMA Network Open.*

eTable 3. Comparisons of Different Generalized Additive Mixed Model Specifications: Black Physicians per Black Population (Negative Binomial)

	Dependent variable:		
	ama.black.docs		
	Model 1 (Variables Only)	Model 2 (+ State-Level Random Effect)	Model 3 (+ Spatial Coordinates)
	(1)	(2)	(3)
% Black population	-2.474	-2.353	-2.339
	(-2.912, -2.036)	(-2.899, -1.807)	(-2.887, -1.791)
	p = 0.000***	p = 0.000***	p = 0.000***
% Illiterate population	-5.548	-5.300	-5.275
	(-7.615, -3.481)	(-7.613, -2.987)	(-7.588, -2.961)
	p = 0.00000***	p = 0.00001***	p = 0.00001***
Log(Terrain ruggedness index)	0.098	0.015	0.009
	(-0.027, 0.223)	(-0.135, 0.165)	(-0.143, 0.160)
	p = 0.126	p = 0.844	p = 0.909
Log(Population density)	-0.200	-0.185	-0.187
	(-0.283, -0.117)	(-0.273, -0.096)	(-0.276, -0.097)
	p = 0.00001***	p = 0.00005***	p = 0.00005***
Number of lynchings, 1896-1905	-0.077	-0.049	-0.049
	(-0.137, -0.018)	(-0.110, 0.012)	(-0.110, 0.012)
	p = 0.011**	p = 0.114	p = 0.114
Log(Median to medical training, Black doctors)	-0.146	-0.090	-0.091
	(-0.200, -0.091)	(-0.150, -0.030)	(-0.151, -0.031)
	p = 0.00000***	p = 0.004***	p = 0.003***
Difference AMD – Census Black doctor count	-0.007	-0.004	-0.003
	(-0.016, 0.001)	(-0.012, 0.004)	(-0.011, 0.005)
	p = 0.090*	p = 0.379	p = 0.396
Constant	-5.924	-6.231	-6.208
	(-6.602, -5.245)	(-7.047, -5.415)	(-7.028, -5.389)
	p = 0.000***	p = 0.000***	p = 0.000***
Observations	310	310	310
Adjusted R^2	0.667	0.796	0.798
Log Likelihood	-507.502	-487.865	-487.852
UBRE	515.807	502.890	502.870
Note:	*p<0.1; **p<0.05; ***p<0.01		

© 2024 Chrisinger BW. *JAMA Network Open.*

eTable 4. Comparisons of Different Generalized Additive Mixed Model Specifications: White Physicians per White Population (Negative Binomial)

	Dependent variable:		
	ama.white.docs		
	Model 1 (Variables Only)	Model 2 (+ State-Level Random Effect)	Model 3 (+ Spatial Coordinates)
	(1)	(2)	(3)
% White population	-0.819	-1.094	-1.154
	(-0.911, -0.726)	(-1.206, -0.982)	(-1.285, -1.024)
	p = 0.000***	p = 0.000***	p = 0.000***
% Illiterate population	-3.232	-3.122	-2.593
	(-3.570, -2.893)	(-3.499, -2.746)	(-3.032, -2.154)
	p = 0.000***	p = 0.000***	p = 0.000***
Log(Terrain ruggedness index)	-0.012	0.007	-0.021
	(-0.039, 0.015)	(-0.023, 0.037)	(-0.061, 0.020)
	p = 0.393	p = 0.651	p = 0.319
Log(Population density)	0.111	0.134	0.112
	(0.085, 0.137)	(0.110, 0.157)	(0.086, 0.138)
	p = 0.000***	p = 0.000***	p = 0.000***
Number of lynchings, 1896-1905	0.023	0.002	0.0001
	(0.008, 0.037)	(-0.011, 0.015)	(-0.013, 0.013)
	p = 0.002***	p = 0.731	p = 0.984
Log(Median to medical training, White doctors)	-0.044	0.010	0.009
	(-0.064, -0.024)	(-0.012, 0.032)	(-0.015, 0.033)
	p = 0.00002***	p = 0.369	p = 0.463
Difference AMD – Census White doctor count	-0.006	-0.006	-0.005
	(-0.007, -0.005)	(-0.007, -0.005)	(-0.006, -0.004)
	p = 0.000***	p = 0.000***	p = 0.000***
Constant	-5.614	-5.874	-5.707
	(-5.797, -5.431)	(-6.078, -5.670)	(-5.945, -5.469)
	p = 0.000***	p = 0.000***	p = 0.000***
Observations	1,518	1,518	1,518
Adjusted R^2	0.876	0.892	0.927
Log Likelihood	-4,917.776	-4,710.761	-4,661.974
UBRE	4,938.118	4,752.101	4,714.090
Note:	*p<0.1; **p<0.05; ***p<0.01		

© 2024 Chrisinger BW. *JAMA Network Open.*

eTable 5. Comparisons of Different Generalized Additive Mixed Model Specifications: Community Representativeness Ratio (CRR; Continuous)

	Dependent variable:		
	CRR		
	Model 1 (Variables Only)	Model 2 (+ State-Level Random Effect)	Model 3 (+ Spatial Coordinates)
	(1)	(2)	(3)
% Illiterate population	-0.219	-0.044	-0.071
	(-0.587, 0.149)	(-0.432, 0.344)	(-0.471, 0.329)
log(Terrain ruggedness index)	0.071	0.035	0.039
	(0.044, 0.098)	(0.005, 0.064)	(-0.002, 0.079)
log(Population density)	-0.001	-0.001	-0.005
	(-0.024, 0.023)	(-0.024, 0.022)	(-0.028, 0.017)
Number of lynchings, 1896-1905	-0.024	-0.015	-0.012
	(-0.037, -0.012)	(-0.027, -0.004)	(-0.023, -0.001)
log(Median distance to training, White doctors)	0.014	0.012	0.020
	(-0.005, 0.032)	(-0.007, 0.031)	(0.001, 0.039)
log(Median distance to training, Black doctors)	-0.017	-0.013	-0.020
	(-0.038, 0.005)	(-0.036, 0.010)	(-0.043, 0.004)
Difference AMD – Census White doctor count	0.001	0.001	0.001
	(0.0003, 0.002)	(0.00004, 0.002)	(-0.0002, 0.002)
Difference AMD – Census Black doctor count	-0.008	-0.007	-0.006
	(-0.012, -0.004)	(-0.011, -0.003)	(-0.009, -0.002)
Constant	0.097	0.175	0.161
	(-0.108, 0.301)	(-0.033, 0.383)	(-0.065, 0.388)
Observations	307	307	307
Adjusted R^2	0.168	0.353	0.386
Log Likelihood	114.280	146.234	152.281
UBRE	-88.753	-109.190	-110.672
Note:	*p<0.1; **p<0.05; ***p<0.01		

© 2024 Chrisinger BW. *JAMA Network Open.*

eTable 6. Associations Between County Characteristics and Differences Between 1906 American Medical Directory and 1910 US Census Occupational Records

	Dependent variable:	
	Difference in Black Doctors	Difference in White Doctors
	(1)	(2)
% Black Population	1.094	
	(0.086, 2.102)	
	p = 0.034	
% White Population		10.099
		(5.270, 14.929)
		p = 0.00005
% Illiterate	-5.716	-19.426
	(-8.984, -2.448)	(-35.370, -3.481)
	p = 0.001	p = 0.018
Ruggedness Index	0.008	0.031
	(-0.0002, 0.017)	(-0.010, 0.072)
	p = 0.057	p = 0.136
Population density (log)	1.414	6.411
	(1.222, 1.606)	(5.462, 7.360)
	p = 0.000	p = 0.000
Number of recent lynchings	0.020	0.039
	(-0.108, 0.148)	(-0.598, 0.677)
	p = 0.762	p = 0.904
Median distance to training	-0.554	-0.134
	(-0.778, -0.330)	(-1.213, 0.944)
	p = 0.00001	p = 0.808
Constant	5.021	-19.345
	(-3.945, 13.988)	(-27.970, -10.719)
	p = 0.273	p = 0.00002
Observations	1,522	1,522
Log Likelihood	-3,822.600	-6,224.098
Akaike Inf. Crit.	7,665.200	12,468.190
Bayesian Inf. Crit.	7,718.478	12,521.470
Marginal R2	0.297	0.168
Note:		$^{*}p<0.1$; $^{**}p<0.05$; $^{***}p<0.01$

© 2024 Chrisinger BW. *JAMA Network Open.*
