# Supplementary material for: US County-Level Variation in Availability and Prevalence of Black Physicians in 1906
Source: JAMA Netw Open. 2024 May 10;7(5):e2410242. doi: 10.1001/jamanetworkopen.2024.10242 (PMC11087833; doi:10.1001/jamanetworkopen.2024.10242)
Supplement: Supplement 2. — Data Sharing Statement [file jamanetwopen-e2410242-s002.pdf]

Data Sharing Statement

Chrisinger. US County-Level Variation in Availability and Prevalence of Black Physicians in 1906. *JAMA Netw Open*. Published May 13, 2024. doi:10.1001/jamanetworkopen.2024.10242

Data
Data available: Yes
Data types: Data (not involving human participants), Data dictionary
How to access data: https://doi.org/10.25446/oxford.24065709.v2
When available: beginning date: 08-31-2023

Supporting Documents
Document types: None

Additional Information
Who can access the data: Data are available to the public.
Types of analyses: Data are available for reuse subject to a Creative Commons license (CC BY 4.0).
Mechanisms of data availability: Data are freely available for download.
